# Nonlinear Analysis of Visually Normal EEGs to Differentiate Benign Childhood Epilepsy with Centrotemporal Spikes (BECTS)

**DOI:** 10.1038/s41598-020-65112-y

**Published:** 2020-05-21

**Authors:** Aarti Sathyanarayana, Rima El Atrache, Michele Jackson, Aliza S. Alter, Kenneth D. Mandl, Tobias Loddenkemper, William J. Bosl

**Affiliations:** 1 Computational Health Informatics Program, Boston Children’s Hospital, Boston, USA; 2 Department of Pediatrics, Harvard Medical School, Boston, USA; 3 Department of Neurology, Boston Children’s Hospital, Boston, USA; 4 School of Nursing and Health Professions, University of San Francisco, San Francisco, USA

**Keywords:** Dynamical systems, Epilepsy, Diagnostic markers, Epilepsy

## Abstract

Childhood epilepsy with centrotemporal spikes, previously known as Benign Epilepsy with Centro-temporal Spikes (BECTS) or Rolandic Epilepsy, is one of the most common forms of focal childhood epilepsy. Despite its prevalence, BECTS is often misdiagnosed or missed entirely. This is in part due to the nocturnal and brief nature of the seizures, making it difficult to identify during a routine electroencephalogram (EEG). Detecting brain activity that is highly associated with BECTS on a brief, awake EEG has the potential to improve diagnostic screening for BECTS and predict clinical outcomes. For this study, 31 patients with BECTS were retrospectively selected from the BCH Epilepsy Center database along with a contrast group of 31 patients in the database who had no form of epilepsy and a normal EEG based on a clinical chart review. Nonlinear features, including multiscale entropy and recurrence quantitative analysis, were computed from 30-second segments of awake EEG signals. Differences were found between these multiscale nonlinear measures in the two groups at all sensor locations, while visual EEG inspection by a board-certified child neurologist did not reveal any distinguishing features. Moreover, a quantitative difference in the nonlinear measures (sample entropy, trapping time and the Lyapunov exponents) was found in the centrotemporal region of the brain, the area associated with a greater tendency to have unprovoked seizures, versus the rest of the brain in the BECTS patients. This difference was not present in the contrast group. As a result, the epileptic zone in the BECTS patients appears to exhibit lower complexity, and these nonlinear measures may potentially serve as a clinical screening tool for BECTS, if replicated in a larger study population.

## Introduction

Childhood epilepsy with centrotemporal spikes, previously known as Benign Epilepsy with Centro-temporal Spikes (BECTS) or Rolandic Epilepsy^1^, is a self-limiting epilepsy that is among the most common forms of epileptic syndromes in childhood, although it is often misdiagnosed or missed entirely. BECTS occurs in 15% of children with seizures between age one and fifteen and most commonly between the ages of seven and ten years^2^. Despite the name, benign forms of epilepsy may be associated with developmental delays and significant neurophysiological deficits. Moreover, they may evolve into more severe epilepsy types leading to severe neurological impairment^3^.

Seizures associated with BECTS can go unrecognized due to their nocturnal, brief and sometimes subtle nature. The seizures occur predominantly during non-rapid eye movement sleep and may last between one and three minutes, or longer. The most common seizure type associated with BECTS is a focal seizure manifesting with altered sensory-motor function of the face, and unilateral facial or arm clonic movements as well as hypersalivation. Evolution into generalized tonic-clonic seizures may also occur. Spiking may occur on the right or left hemisphere in variable frequencies and magnitudes, but diagnosis continues to be based on clinical assessment. On occasion children with BECTS may also have normal electroencephalogram (EEG)^2,4^.

Although BECTS often remits in adolescence^5^, it can lead to neurocognitive impairments in memory and phonological processing^6^, language^7^, fine motor skills^8^, and may be associated with a lower IQ^9^ Patients are at risk for developing behavioral problems and academic difficulties especially with early onset^10,11^. The relationship between BECTS and cognitive development has not yet been well studied.

### Methodological rationale

EEG has long been used in neurology as the primary tool for detecting and classifying epilepsy. The use of computational methods to extract information from EEG signals to be used to detect emerging disease is less well developed. Several recent studies have used quantitative EEG analysis to compute EEG measures that may be used to detect attention deficit/hyperactivity disorder^12^, autism spectrum disorder^13,14^, treatment response for depression^15^, and absence epilepsy^16^ In addition to screening or disease diagnosis, EEG-based measures could be used to monitor disease progression or treatment response, or to predict clinical outcomes. Associating brain electrodynamics with levels of epileptogenicity may also provide an opportunity to explore early interventions, and monitor treatment response longitudinally. The results reported in this study are specific to BECTS, but the methods used may be applicable to other seizure and epilepsy types. The aim of this study was to identify features computed from EEG signals that might be further explored in larger studies to differentiate BECTS patients from patient without BECTS while patients are awake, potentially supplementing or streamlining current diagnostic evaluations when BECTS is suspected.

Previous research has demonstrated that high frequency discharges associated with the epileptic process are accompanied by a loss in complexity in the signals from the epileptogenic area^17^. Moreover, various measures of nonlinear dynamics have been computed from EEG time series in order to detect changes immediately prior to the onset of seizures or epileptiform discharges^18,19^. Using a single measure of signal complexity to characterize time series from intracranial electrodes, the epileptogenic region in focal epilepsy patients was located during interictal periods, in agreement with pre- and post-surgical results obtained by traditional measurements. In another study of intracranial recordings, it was demonstrated that nonlinear cross-correlation could be used to find entrainment between pairs of signals, which localized regions of focal epileptic activity^20,21^. Recently, machine learning was applied to nonlinear signal features derived from EEG measurements taken as early as three months of age to predict infants who later developed autism spectrum disorder from those that did not^13^.

In our analysis, we compute several nonlinear measures on multiple frequency bands or scales. One approach to computing a series of nonlinear measures is based on a statistical analysis of recurrence plots. Recurrence quantitative analysis or RQA is an approach to analyzing time series that derives quantitative measures such as entropy from recurrence plots that are projections of high-dimensional phase space onto two dimensional plots^22–25^. Recent research has demonstrated the relevance of RQA to epilepsy detection^26–28^. Recurrence quantitative analysis has been used for early seizure detection by distinguishing ictal and interictal entropy states and recently for differentiating children with autism spectrum disorder from typically developing children^29^. Our work builds on these results by developing a general approach to computing nonlinear dynamical features from EEG signals from a clinically well characterized epilepsy patient population, and evaluate these results to determine sensor location and measures that are distinctly different in BECTS patients even while awake.

## Methods

### Study population

This study was approved by the IRB at Boston Children’s Hospital (IRB-P00001945). The study design was a retrospective de-identified record review, and therefore this study was deemed to be exempt from consent by the Boston Children’s Hospital Institutional Review Board. The research was performed in accordance with the guidelines and regulations of the Institutional Review Board at Boston Children’s Hospital and all applicable government regulations, including the Helskinki Guidelines.

**Figure 1:**
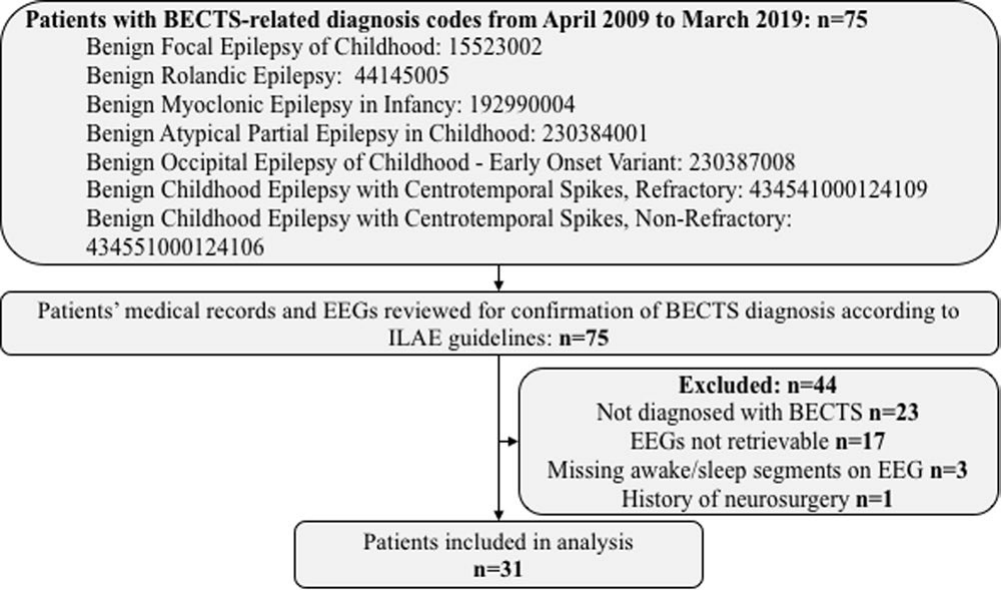
Inclusion/exclusion tree for patients diagnosed with BECTS.

We retrospectively reviewed all electronic medical records of patients with BECTS diagnosis codes admitted to the long-term monitoring unit at Boston Children’s Hospital (BCH) between April 2009 and March 2019. Thirty one patients with BECTS (13 female, median age 9.5 years) were retrospectively selected from the BCH Epilepsy Center database after BECTS was confirmed by clinical history and the EEG findings of an experienced neurologist. As per the International League Against Epilepsy (ILAE) guidelines, patients were diagnosed with BECTS if their EEGs showed frequent drowsiness and sleep activated high amplitude centrotemporal spikes or sharp-and-slow wave complexes that may be unilateral or bilateral^1^. These spikes present with maximum negativity in central and temporal electrodes and maximum positivity in the frontal electrodes. For the BECTS cohort, we excluded patients who were not diagnosed with BECTS, whose EEG data were not retrievable and/or those who had a history of neurosurgery. Figure 1 shows the detailed inclusion and exclusion tree for the cohort of patients with BECTS.

**Figure 2:**
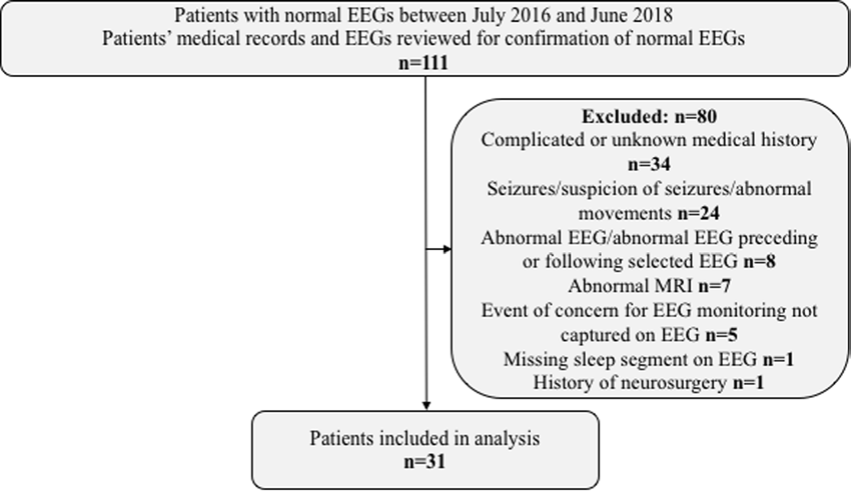
Inclusion/exclusion tree for the contrast cohort.

For comparison purposes, thirty-one patients (10 female, median age 10 years) were selected for a contrast group, using records from July 2016 to June 2018. The contrast group patients had no form of epilepsy and had a normal EEG, based on clinical chart review. We excluded patients from the contrast cohort who had seizures, ongoing suspicion of seizures after EEG was completed, were treated with epilepsy medications, an abnormal EEG preceding or following the EEG that was analyzed, an abnormal MRI, irretrievable EEG data and/or other related medical conditions. Figure 2 depicts a detailed inclusion and exclusion tree for the selection of the group of contrast patients Table 1 shows the demographic and clinical characteristics of the BECTS patients included in this study. Table 2 shows the comorbidities and reasons an EEG was taken for the patients in the contrast cohort.

**Table 1:** Demographic and Clinical characteristics of patients with BECTS at the time of EEG (n = 31).

Demographic Characteristics	Median Years, (Range, IQR)
Female, n (%)	13 (41.9)
Median Age at EEG	9.5 (11.1, 3.6)
Median Age at First Seizure	7.9 (9.4, 4.6)
**Ethnicity**	**n (%)**
Not Hispanic or Latino	20 (64.5)
Unknown	5 (16.1)
Hispanic or Latino	4 (12.9)
Not Reported	2 (6.5)
**Race**	**n (%)**
White	22 (71.0)
Unknown	4 (12.9)
Black or African American	3 (9.7)
Not Reported	1 (3.2)
Asian	1 (3.2)
**Clinical Characteristics**	**n (%)**
Seizure Types - Movement*	
Motor	30 (96.8)
Unknown	1 (3.2)
Focal Onset - Level of Awareness*	
Impaired Awareness	7 (22.6)
Aware	5 (16.1)
Unknown	4 (12.9)
Comorbidities*	
None	19 (61.3)
Developmental/Psychiatric Disorders	8 (25.8)
ADHD	3 (9.7)
Unspecified Developmental Delay	2 (6.5)
Anxiety	2 (6.5)
Intellectual Disability	1 (3.2)
Other Medical Conditions*	7 (22.6)
Gastrointestinal Disorders	2 (6.5)
Respiratory Disorders	2 (6.5)
Skin Disorders	2 (6.5)
Hearing Impairment/Deafness	2 (6.5)
Urinary Tract Disorders	1 (3.2)
Genital Disorders	1 (3.2)
Sleep Disorders	1 (3.2)
Obesity	1 (3.2)
Motor Disorder	1 (3.2)
Prescribed Home AEDs Prior to EEG Hospital Admission*	
None	18 (58.1)
Diazepam (Diastat)	4 (12.9)
Levetiracetam	2 (6.5)
Oxcarbazepine	2 (6.5)
Valproic Acid/Valproate	2 (6.5)
Zonisamide	2 (6.5)
Ethosuximide	1 (3.2)
Lacosamide	1 (3.2)
Topiramate	1 (3.2)
Clobazam	1 (3.2)
**AEDs Administered During EEG Hospital Admission**	
None	26 (83.9)
Levetiracetam	1 (3.2)
Oxcarbazepine	1 (3.2)
Topiramate	1 (3.2)
AEDs Withheld	1 (3.2)
Unknown	1 (3.2)
**Other Seizure Treatments Administered During EEG Hospital Admission**	
Ketogenic Diet	1 (3.2)
**MRI Conducted Within 6 Months Prior/Post EEG**	
Yes	25 (80.6)
No	6 (19.4)
**MRI Findings**	
Normal	22 (71.0)
Cyst	2 (6.5)
Degeneration	1 (3.2)
**MRI Lesion Location**	
Corpus Callosum	1 (3.2)
Hippocampus	1 (3.2)
Temporal Lobe - Mesial Temporal	1 (3.2)
Temporal Lobe - Neocortical or Unspecified Temporal	1 (3.2)
**CT Conducted Within 6 Months Prior/Post EEG**	
No	25 (80.6)
Yes	6 (19.4)
**CT Findings**	
None	5 (16.1)
Unknown/Not Reported	1 (3.2)

*Patients may have been represented in more than one category and numbers, therefore, do not add up to 31 (100%). Values are n (%) unless otherwise indicated.

**Abbreviations: EEG:** Electroencephalogram**, BECTS:** Benign Partial Epilepsy of Childhood with Centro-Temporal Spikes, **MRI:** Magnetic Resonance Imaging.

**Table 2:** Clinical characteristics of patients in the contrast cohort including the clinical reason for an EEG as well as their comorbidities.

Clinical Characteristics of Contrast Cohort	n(%)
Event of Concern for EEG	
Staring	11 (35.5)
Abnormal Behavior or Body Movements	10 (32.3)
Headache	9 (29.0)
Eye-Rolling	8 (25.8)
Bilateral Extremity Jerk or Other Jerking	10 (32.3)
Decreased Responsiveness/Unresponsiveness	11 (35.5)
Dizziness	4 (12.9)
Anxiety	3 (9.7)
Behavioral Arrest	3 (9.7)
Confusion or Altered Mental Status	5 (16.1)
Unprovoked Laughter	2 (6.5)
Developmental Delay	3 (9.7)
Head Drooping	1 (3.2)
Night Terrors	1 (3.2)
Transient Hypotonia	1 (3.2)
Vomiting	1 (3.2)
Unspecified Spells	1 (3.2)
**Comorbidities**	**n(%)**
**Development/ Psychiatric Disorders**	**13 (41.9)**
Autism	5 (16.1)
Anxiety	5 (16.1)
ADHD	4 (12.9)
Behavioral/Emotional Disorders	3 (9.7)
Developmental Language Delays	3 (9.7)
Depression	2 (6.5)
Psychosis	2 (6.5)
Trisomy 21 Global Developmental Delay	2 (6.5)
Developmental Delay/Regression	2 (6.5)
Developmental Dyslexia	1 (3.2)
Dyspraxia	1 (3.2)
**General Medical Conditions**	**11 (35.5)**
Gastrointestinal Disorders	2 (6.5)
Chronic Lower Respiratory Disorders	2 (6.5)
Cardiovascular Disorders	2 (6.5)
Congenital Non-Neurologic Malformation	1 (3.2)
Musculoskeletal Disorders	1 (3.2)
Malnutrition/Eating Difficulties	1 (3.2)
Skin Disorders	1 (3.2)
Endocrine Disorders	1 (3.2)
Hematologic conditions	1 (3.2)

### Data collection

A Board-certified child neurologist/neurophysiology fellow reviewed EEGs of patients with BECTS diagnosis codes. Review confirmed the diagnosis of BECTS from EEGs and at least 30 s segments were taken while the patient awake such that they included no spikes during the patient’s awake EEG. Natus NeuroWorks software version 8 was used to view EEGs and each sample was collected on 19 channels located according to the standard 10–20 system. For the contrast group patients, the child neurologist/neurophysiology fellow confirmed that the EEGs were normal based on visual inspection. After review, EEG recordings were saved in an open format (EDF) file on a research server for analysis by research assistants. Thirty second EEG segments were randomly selected for analysis from the saved files by the signal analysis code from both the BECTS cases and the contrast group, during the awake periods. No other filtering was performed on the EEG signals.

### Signal processing

Dynamical values were computed from the EEG signals including multiscale sample entropy and several others derived from a multiscale approach to Recurrence Quantitative Analysis. In principle, RQA is capable of detecting significant state changes in a dynamical system, which suggests that it may be appropriate for detecting BECTS. All nonlinear features used in this study were computed on multiple frequency bands using publicly available methods and tools. The multiscale nonlinear measures that were particularly useful were sample entropy (SampE), trapping time (TT), and the maximum and mean diagonal line length of the recurrence plot (Lmax and Lmean, respectively). Table 3 provides a brief description of these measures and the currently understood meaning of these computed features^30^.

**Table 3:** Definitions of the nonlinear measures computed on a 30-second EEG interval of a patient’s “awake” EEG.

Nonlinear Invariant Variable	Description
Sample Entropy	Sample entropy measures the complexity of a time-series. It is precisely the negative natural logarithm of the conditional probability that two sequences similar for *m* points remain similar at the next point, where self-matches are not included in calculating the probability^43^. Its computation is based on approximate entropy, but reduces bias and relative consistency, while being largely independent of signal length. A lower value of sample entropy indicates more self-similarity in a signal.
Trapping time	Trapping time is an estimate of the time that a system will remain in a given state, such as the length of transition states, as opposed to the time for the transition to take place.
Max line length (Lmax)	Lmax is related to the largest Lyapunov exponent of a chaotic signal, which is a dynamic complexity measure that describes the divergence of trajectories with small differences in initial states^44^. The higher Lmax, the greater sensitivity to initial conditions, and the less predictable signal behavior.
Mean line length (Lmean)	The time that two segments of the recurrence plot trajectory are close to each other, and can be interpreted as the mean prediction time of the signal, a measure of chaos or divergence from an initial point

### Required software packages

All computations performed for this study were carried out using standard python packages, including numpy and scipy, and other publicly available packages listed here:

eegtools (for reading edf files): https://github.com/breuderink/eegtools

pywavelets (for wavelet decomposition): https://pywavelets.readthedocs.io

pyrqa (RQA calculations): https://pypi.python.org/pypi/PyRQA/

nolds (Sample entropy and DFA calculations): https://pypi.python.org/pypi/nolds

scikit-learn (feature ranking and machine learning): http://scikit-learn.github.io/stable

### EEG data availability

The methods described can be applied to any set of EEG data together with diagnostic labels. The specific participant data used for our study was consented specifically for use by researchers affiliated with the Department of Neurology at Boston Children’s Hospital. For this reason, the raw EEG data cannot be released publicly.

## Results

The aim of this study was to identify EEG based measures that can differentiate between patients with BECTS and those without BECTS, and may be explored further in larger studies as possible digital biomarkers. We found that there was a difference between several multiscale nonlinear measures of awake BECTS patients and awake patients without BECTS in our contrast group, while visual EEG inspection did not reveal any distinguishing features. In addition, we found that in spite of the lack of visually appreciable signs of BECTS in the awake EEG, as determined by a board-certified child neurologist / neurophysiology fellow, there was a difference between the same nonlinear measures in the centrotemporal region of the brain versus the rest of the brain. This difference was not present in the contrast group.

### Differentiating between patients with BECTS and those without

**Figure 3:**
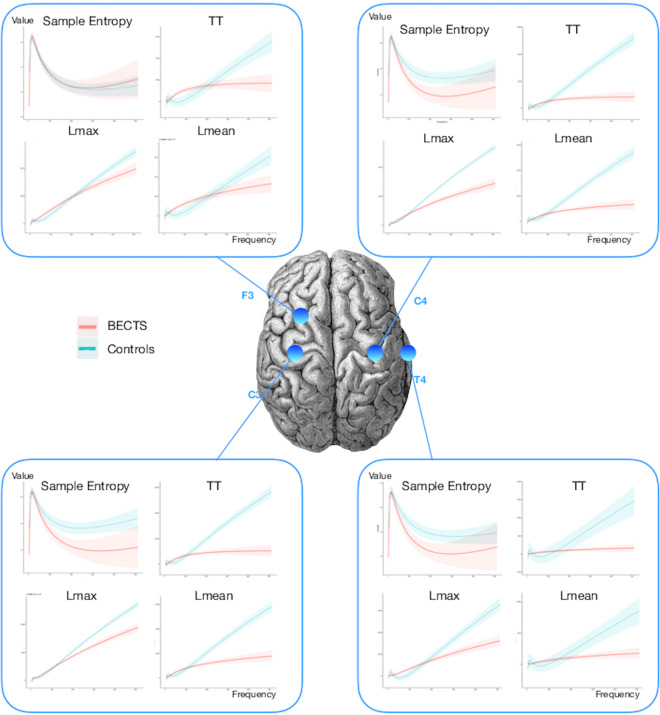
The multiscale graphs of sample entropy, trapping time (TT), maximum (Lmax) and mean (Lmean) diagonal line length from the recurrence plot for a sub-selection of sensors labeled as per the standard 10–20 EEG system. The 95% confidence intervals of each curve are shown in a sheer corresponding color. There is a visual distinction between the curves representing BECTS patients (red) and patients without BECTS (blue). Moreover, the distinction between the curves is greater in the centrotemporal region than in the frontal region. Sensor F3 is from the left frontal region of the brain, C3, C4 and T4 are in the centrotemporal regions. Brain image by: Paul Broca, Memoires d’Anthropologie, 1877. Credit: Wellcome Collection. Creative Commons license 4.0. https://wellcomecollection.org/works/fnzzu2p8.

Figure 3 illustrates several key differences between the interictal electrodynamics of a patient’s brain with BECTS versus a patient’s brain without BECTS, on a selection of sensors. We computed the nonlinear measures from a 30-second interval of an EEG taken when the patient was in an awake state. These EEGs contained no visual signs of epileptiform activity. We created multiscale graphs to plot the measures over different frequency bands. Each curve was computed by aggregating the measure for each sensor over each cohort of patients: BECTS or the contrast group. For sample entropy, trapping time, Lmax and Lmean, the differences between BECTS and the contrast group increases for higher frequencies. Moreover, some sensors showed more dramatic distinction than others. The 95% confidence intervals are shown by the shaded borders in the corresponding color. For some features, the confidence interval was much larger than others.

Statistically significant group differences were found for several nonlinear features, including the largest Lyapunov exponent, Lmax (p < 10e-50 for all frequency bands). These results suggest that although BECTS patients are most likely to seize at night, that differences can be detected in EEG-derived nonlinear measures even when patients are awake.

### Irritative regions of the brain in patients with BECTS

**Figure 4:**
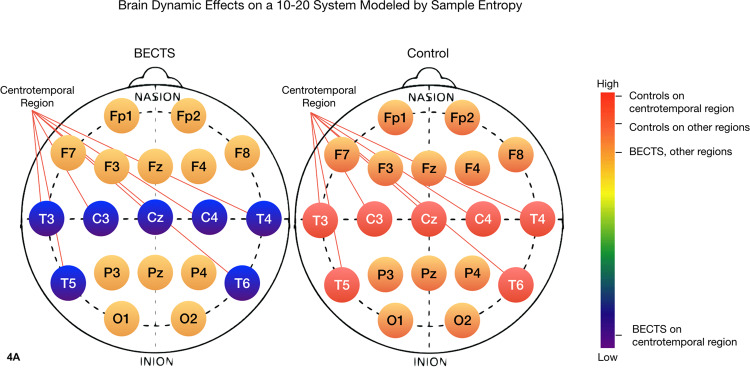
Heuristic heatmaps on the 10–20 EEG system, chromatically representing the area under the curve of a multiscale curve for a nonlinear invariant, on the awake brain of a BECTS patient (left) and a patient without BECTS (right). Sensors C3, C4, Cz, T7, T8, P7 and P8 are placed on the centrotemporal region of the brain, and thus cover the epileptogenic zone for a patient diagnosed with BECTS. Images created in part using R version 3.4.3 (2017–11–30), http://cran.r-project.org/src/base/R-3/R-3.4.3.tar.gz. The sample entropy of the BECTS epileptogenic region is lower than the sample entropy of a patient without epilepsy. Moreover, the sample entropy within the brain of a patient with BECTS is different between the centrotemporal region and the rest of the brain. This indicates that the centrotemporal region of a BECTS brain, i.e. the epileptogenic region of the brain, has more self-similarity within the EEG signal, which is consistent with the notion that seizures are related to neural synchronization. 1-way ANOVA: F(df=3) = 5.2, p < 10^−3^.

**Figure 5:**
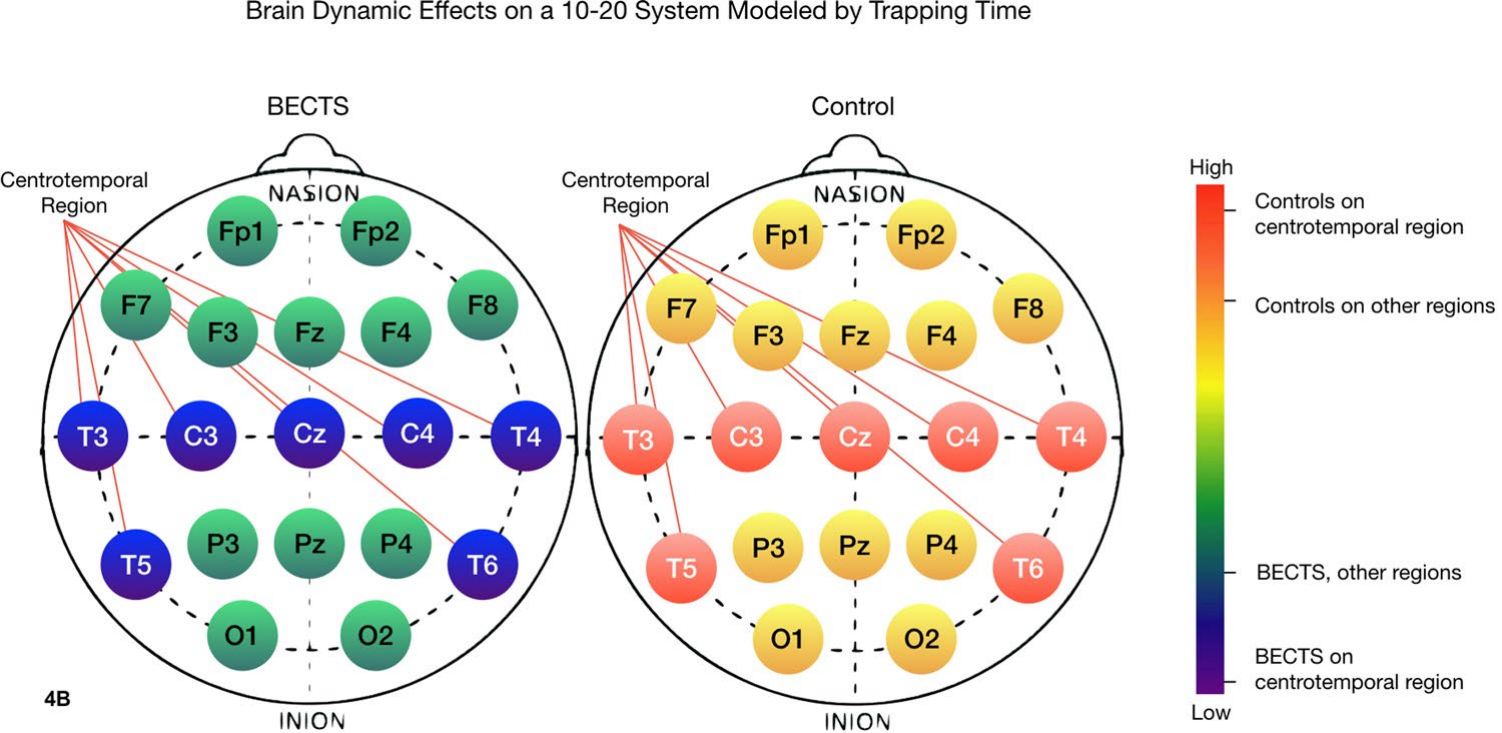
The trapping time of the BECTS brain is overall lower than the trapping time of the contrast cohort brain. There is also a distinction between the trapping time of the centrotemporal region on the BECTS brain versus the contrast group. The low trapping time visible in the BECTS brain indicates more transition between states, and less stability in the brain electrodynamics. 1-way ANOVA: F(df=3) = 63.3, p < 2×10^−16^.

**Figure 6:**
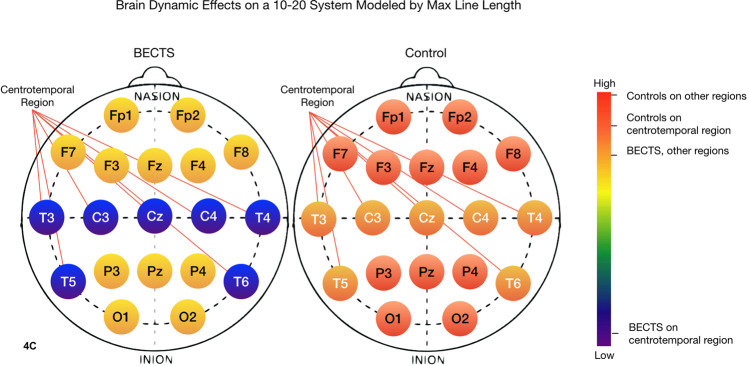
The maximum diagonal line length of the recurrence plot (Lmax) on the centrotemporal region of the BECTS brain is much lower than for the contrast group brain, as well as for the other regions of the BECTS brain. Moreover, the difference between Lmax on the centrotemporal region of the brain versus the rest of the brain is much larger for patients with BECTS versus patients without. Lower Lmax indicates less chaos and less complexity in the electrodynamics. Past studies have found that lower Lmax is associated with slowing of brain electrical activities, and it has been associated with other neural pathologies^42^. 1-way ANOVA: F(df=3) = 43.9 p < 2×10^−16^.

**Figure 7:**
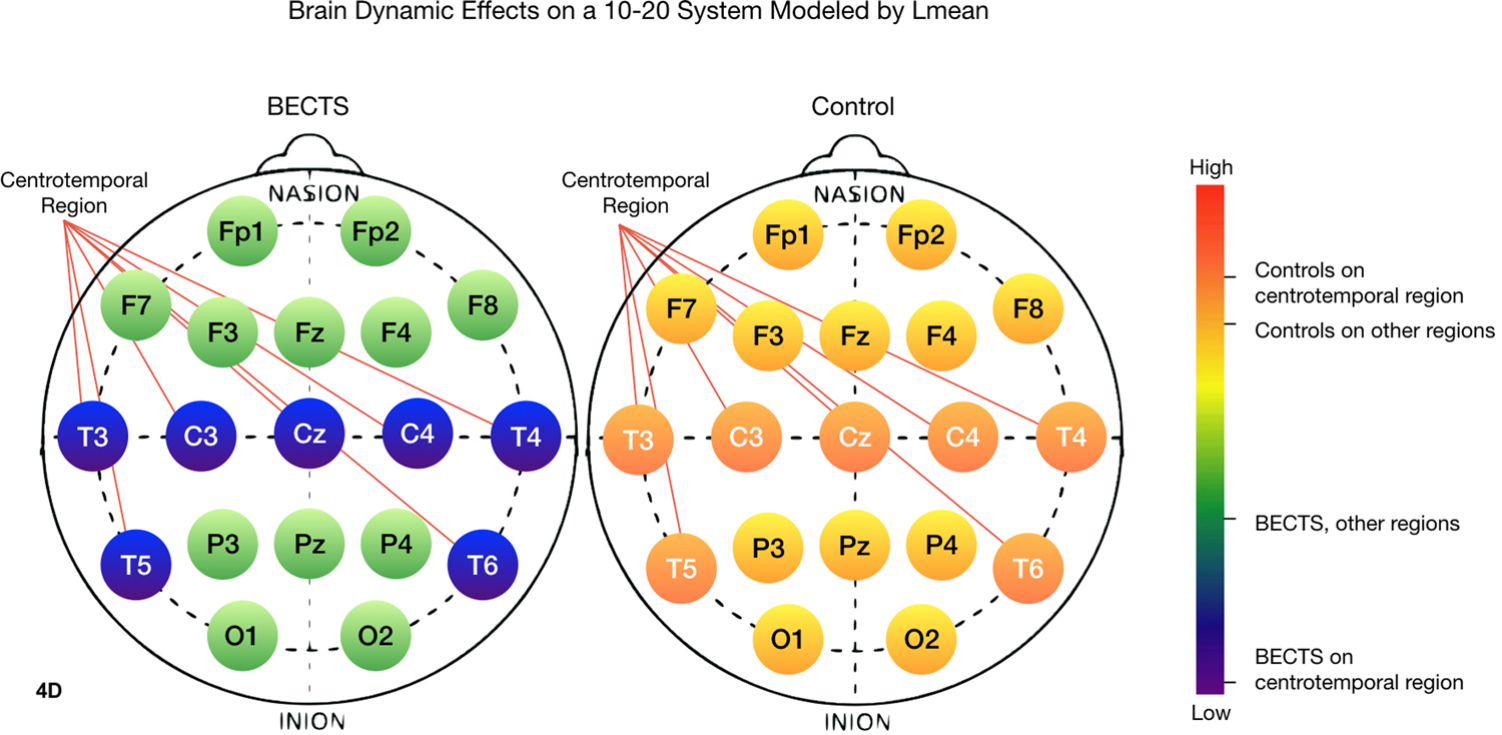
The mean diagonal line length of the recurrence plot (Lmean) is lower for the BECTS brain than for the contrast group. On the BECTS brain, Lmean is lower for the centrotemporal region than for the rest of the brain, indicating less chaos and complexity in the electrodynamics. 1-way ANOVA: F(df=3) = 46.7, p < 2×10^−16^.

BECTS seizures are localized to the centrotemporal region of the brain. From a dynamical perspective, a digital biomarker for BECTS, as opposed to other forms of epilepsy, would be expected to identify pathological activity within the centrotemporal region in particular. Figures 4–7 chromatically represent the area under the multiscale curve for each nonlinear measure. The multiscale curve is computed across two categories of patients (BECTS and the contrast group) and two categories of sensors, centrotemporal (CT) and all others (non-CT). These heatmaps illustrate the differences between the interictal nonlinear dynamics of an awake patient with BECTS versus a patient without BECTS for 4 nonlinear measures: sample entropy, trapping time, Lmax and Lmean. We found that the centrotemporal region of the BECTS brain has distinct differences to the other regions. Specifically, the distinction between the centrotemporal region on BECTS relative to the remainder of the brain is greater than the difference between the centrotemporal region on a patient without BECTS relative to the non-centrotemporal sensors.

Color bars on the right side of Figs. 4–7 show the mean value of the nonlinear measure for each of the four regions: CT and non-CT sensors for the two groups, BECTS and the contrast cohort. A one-way between regions ANOVA was conducted to compare the effect of region on nonlinear values. There was a difference between sensors at the p < 0.05 level for all nonlinear measures, with TT, Lmax, and Lmean being the most significant [SampE: F(3) = 4.94, p < 1×10^−3^, TT: F(3) = 63.3, p < 2×10^−16^, Lmax: F(3) = 43.9, p < 2×10^−16^, Lmean: F(3) = 47.7, p < 2×10^−16^]. The last three are notably significant, suggesting that the corresponding nonlinear measures may be good candidates for a larger clinical biomarker study with an independent test set.

## Discussion

Two related findings are reported based on this study. First, several nonlinear measures derived from EEGs are clearly different in the centrotemporal region of BECTS patients when compared to the same region in patient without BECTS. Because all results are from awake patients, this suggests a possible method to detect BECTS in an awake patient. Secondly, these measures clearly distinguish the centrotemporal and extra-centrotemporal regions in the same patient, suggesting that these nonlinear measures may tentatively be useful for detecting the epileptogenic zone. These results are explored in more detail below.

In our analysis, several nonlinear features computed from EEG signals were different between awake patients in the contrast and BECTS groups. The most important features included sample entropy, TT, Lmax, and Lmean, across multiple frequency bands. The current understanding of these measures are typically in the context of physical systems, from which most dynamical systems research derives. It is not entirely clear whether each of these measures are independent metrics of nonlinear system dynamics. The neurophysiological meaning of these will require considerable focused research. We offer an interpretation of our current results that may be useful for understanding our findings in the context of epilepsy.

High frequency oscillations (HFOs), defined as ripples or fast ripples with frequencies greater than 70 Hz, have emerged over the past two decades as potential biomarkers for epilepsy^31–33^, and more specifically for mapping the epileptic zone prior to surgical resection^34–36^. HFOs are of particular interest because they appear to be present during interictal periods, before seizure onset, and during seizures^31^. HFOs continue to be studied as potential biomarkers for the epileptogenic zone in focal epilepsies^35^, particularly in the setting of pre-surgical mapping^34,36^. Scalp-recorded HFOs have been found in children with continuous spike-waves during slow-wave sleep^37,38^. These results are of interest to the present study because the ubiquitous presence of HFOs in epilepsy suggests that the epileptic regions exhibit different dynamics than the non-epileptic zone. For example, HFOs have been associated with lower sample entropy in the epileptic zone^39^. We speculate that the functional dynamics of neural networks in the epileptic zone exhibits lower complexity, which results in lower sample entropy, and other complexity measures reported here. The lower complexity is also associated with the occurrence of HFOs. That is, HFOs are a symptom of the altered nonlinear dynamics of lower complexity.

### Scalp location of most significant features

Seizures represent a synchronization process in which large regions of neurons are entrained and fire in a coordinated manner. The resulting synchronous spiking neurons result in large amplitude, highly periodic activity seen on the EEG trace. This activity is less complex and more uniform that non-seizure activity. We speculate that seizures are more likely to occur when the epileptic brain undergoes a pathological phase shift to a dynamical regime that allows the hypersynchronization of a seizure to occur. A lower level of complexity is often a sign of disease in biological systems^23^.

Sample entropy and Lmax differ primarily in the epileptic zone in the BECTS patients, while Lmean and TT appear to differ from the contrast group in all scalp regions, though the differences are greater in the centrotemporal zone. More recently, functional and also minor structural abnormalities were detected on imaging studies in the centrotemporal area, such as cortical thickness, in patients with BECTS, potential reflecting our electrophysiological findings^40,41^. Although lateralization of centrotemporal spikes (CTS) was observed, it did not appear to be correlated with fMRI activation; fMRI activation was correlated with neuropsychological differences. Our study can be extended in the future by reviewing the EEG records to assess for CTS frequency and lateralization, then evaluating correlation between actual spiking location and frequency and the nonlinear measures that we found to differ in the BECTS patients.

Our findings suggests that Lmean and TT are measuring brain dynamics that may extend beyond the epileptic zone in ways that are not yet understood.

### Challenges

These results need to be interpreted in the setting of the data acquisition. Our results are exciting in that they suggest a tentative novel set of EEG measures that might be further explored as potential biomarkers for BECTS. However, the sample size is small, thus generalization of results may be limited at this stage. Here we describe group difference, but details for individual performance assessment will require further assessment. In an attempt to minimize confounding, we selected a relatively homogeneous clinical population without clear lesions on MRI, and without spikes in EEG samples. Due to the patient identification approach, we cannot rule out selection bias, however patient selection also helped us reduce confounders. Lastly, the contrast group members were patients who underwent EEG for clinical purposes, and these patients did not have epilepsy on chart review. However, as an EEG was performed there may have been some clinical suspicion of a seizure disorder. These features of the contrast group should have reduced differences, not increased differences between both groups. Our results are a first step of differentiating BECTS and normal patients, but evaluation of potential cut off values and prospective assessment are next important steps.

### Conclusions: clinical implications and future research

Our results indicate that the contrast cohort had higher sample entropy, trapping time, Lmax, and Lmean values in all brain regions when compared to BECTS patients. Each of these measures some aspect of nonlinear dynamics or chaos in a system. Although a full discussion of nonlinear dynamical systems is well beyond the scope of this paper, we offer some neurobiological interpretations that may suggest future clinical and basic research. We speculate that neural populations with higher levels of chaotic activity may be able to synchronize only transiently and thus have a lower chance for runaway synchronization that results in a seizure. Thus we would expect to see lower sample entropy in a BECTS patients than in patients without epilepsy. Our results are consistent with this hypothesis. Similarly, trapping time is an estimate of the time that a system will remain “trapped” in a given state. Though related to entropy, it is a different measure. Higher trapping time indicates more time in a given state and less opportunity for neuronal synchrony. Lmax describes the divergence of trajectories starting at similar initial states and is related to the more commonly reported Lyapunov exponent. A higher Lmax value indicates diverging trajectories and thus less time for synchronous entrainment leading to seizures. Lastly, Lmean describes the time that two recurrence plot trajectories are close to each other. This is related to Lmax and has a similar implication: less opportunity for synchrony and thus we would expect to see lower mean line length values in BECTS patients. Our results are consistent with this hypothesis. Future testing on larger populations with both training and test sets will be needed to determine accuracy, sensitivity, specificity, and other statistical parameters required for future clinical use. Similarly, larger cohorts with BECTS cases and other epilepsies may enable deeper exploration of the neurodynamics of epilepsy.
